# Calcium oxalate crystals and oxalate induce an epithelial-to-mesenchymal transition in the proximal tubular epithelial cells: Contribution to oxalate kidney injury

**DOI:** 10.1038/srep45740

**Published:** 2017-04-07

**Authors:** Marcia Bastos Convento, Edson Andrade Pessoa, Edgar Cruz, Maria Aparecida da Glória, Nestor Schor, Fernanda Teixeira Borges

**Affiliations:** 1 Department of Medicine, Nephrology Division, Universidade Federal de São Paulo (UNIFESP), São Paulo, SP, Brazil; 2 Postgraduate Program, Health Sciences, CBS, Universidade Cruzeiro do Sul, São Paulo, Brazil

## Abstract

TGF-β1 is the main mediator of epithelial-to-mesenchymal transition (EMT). Hyperoxaluria induces crystalluria, interstitial fibrosis, and progressive renal failure. This study analyzed whether hyperoxaluria is associated with TGF-β1 production and kidney fibrosis in mice and if oxalate or calcium oxalate (CaOx) could induce EMT in proximal tubule cells (HK2) and therefore contribute to the fibrotic process. Hyperoxaluria was induced by adding hydroxyproline and ethylene glycol to the mice’s drinking water for up to 60 days. Renal function and oxalate and urinary crystals were evaluated. Kidney collagen production and TGF-β1 expression were assessed. EMT was analyzed *in vitro* according to TGF-β1 production, phenotypic characterization, invasion, cell migration, gene and protein expression of epithelial and mesenchymal markers. Hyperoxaluric mice showed a decrease in renal function and an increase in CaOx crystals and Ox urinary excretion. The deposition of collagen in the renal interstitium was observed. HK2 cells stimulated with Ox and CaOx exhibited a decreased expression of epithelial as well as increased expression mesenchymal markers; these cells presented mesenchymal phenotypic changes, migration, invasiveness capability and TGF-β1 production, characterizing EMT. Treatment with BMP-7 or its overexpression in HK2 cells was effective at preventing it. This mechanism may contribute to the fibrosis observed in hyperoxaluria.

Calcium oxalate (CaOx) crystals are the major crystalline composition of kidney stones, as being CaOx crystallization is influenced by the concentration of calcium and oxalate in the kidney tubules^1^. Both CaOx crystals and oxalate (Ox) ions stimulate localized injury and inflammation in the kidneys, particularly near the tubules. Oxalate nephropathy is characterized by tubular crystalline deposits of calcium oxalate and can lead to acute and chronic tubular injury, interstitial fibrosis, tubulointerstitial inflammation, and progressive renal insufficiency nephropathy^2^. The underlying mechanism of fibrosis induced by oxalate is poorly understood but may involve epithelial to mesenchymal transition (EMT).

EMT plays a crucial role in both physiologic and pathologic conditions, such as embryogenesis, cancer, and fibrosis. This transition process describes a phenotypical change induced in epithelial cells, which in the setting of tissue injury gives way to a cell type termed the myofibroblast; these cells are characterized by the dissolution of tight junctions, loss of cell-cell adhesion, loss of apico-basal polarity, reorganization of the actin cytoskeleton, and increased motility, extracellular matrix protein synthesis, proliferation, and invasiveness^3^,^4^,^5^. The cells acquire mesenchymal characteristics, including down-regulation of epithelial markers E-cadherin, cytokeratin, and tight junction protein zonula occludens-1 (ZO-1), resulting to disintegration and loss of the cell contact. In contrast, the cells undergoing EMT up-regulate mesenchymal markers, such as vimentin and smooth muscle α-actin^6^,^7^,^8^.

TGF-β1 is a key profibrotic cytokine found in the kidney and in other solid organs^9^. It induces numerous cellular responses by acting as a central orchestrator of development, wound healing, fibrosis, and cancer. Macrophages are the main cell involved in the cytokine production; it has been shown that macrophages exposed to CaOx stimulate TGF-β1 production and induce EMT in distal tubular epithelial cells^10^.

The proximal tubule is the main segment of the nephron that is exposed to oxalate. Additionally, the proximal tubular epithelial cells are capable of taking up CaOx crystals through endocytosis and producing TGF-β1^11^. Therefore, it is reasonable to suggest that when exposed to Ox or CaOx, the proximal tubule may participate in extracellular matrix deposition in peritubular fibrosis through EMT.

The origin of the fibroblasts involved in renal fibrosis has been previously demonstrated in chronic kidney disease, with 50% arising from local resident fibroblasts through proliferation, 35% from myofibroblast differentiation in the bone marrow, 10% from the endothelial-to-mesenchymal transition program, and 5% from the epithelial-to-mesenchymal transition program^4^,^12^. Nevertheless, the role of Ox and CaOx in the production of TGF-β1 and in the induction of EMT into proximal tubule cells and the kidney cortex have not yet been determined.

Bone morphogenic protein-7 (BMP-7) has emerged as a key antifibrotic cytokine in the kidney that prevents fibrosis and antagonizes the effects of TGF-β1^13^. It is a member of the transforming growth factor-β1 (TGF-β1) superfamily, which plays a crucial role in renal development^14^,^15^. In the adult kidney, BMP-7 expression can be detected in tubular epithelial cells and also in podocytes^16^,^17^. Its expression significantly decreases during acute renal injury, and the administration of exogenous rhBMP-7 accelerates the repair of the injured kidney, suggesting that it plays a role in the maintenance of kidney homeostasis^18^,^19^,^20^,^21^.

The goal of the present study was to analyze the effect of Ox and CaOx both *in vivo* and *in vitro* as well as their ability to induce EMT *in vitro*. The renal cortex of hyperoxaluric mice revealed CaOx crystal deposition and increased production of TGF-β1 and collagen I and III. Both Ox and CaOx induced EMT in the proximal tubular epithelial cells through the expression of TGF-β1. Treatment with BMP-7 and its overexpression in tubular cells conferred resistance to the effects of Ox and CaOx.

## Methods

### *In vivo* assays

The experimental protocol was approved by the Ethics Committee of the Universidade Federal de São Paulo (UNIFESP - protocol number 2012/05412), also in agreement with the Brazilian guidelines for scientific animal care and use^22^,^23^.

#### Experimental groups

C57Bl/6 mice were divided into the following groups: a control group receiving water *ad libitum* for 60 days; an HPL group that received 5% trans-4-hydroxy-L-proline for 30 or 60 days *ad libitum*; and an ETG group receiving ethylene glycol (1%) *ad libitum* for 30 or 60 days, all Sigma reagents (Sigma-Aldrich, MO, USA). At the end of the experimental protocol, the animals were kept for 24 hours in metabolic cages for urine collection and were then sacrificed using a toxic dose of anesthetic (ketamine and xylazine). The kidneys were removed, and the cortex was collected for histological analysis. Blood was obtained for the urea and creatinine analyses. The urine volume was measured and used to assess the Ox, number of crystals, and creatinine clearance. The experimental protocol was repeated 3 times at different moments. N = 5 for each group.

#### Urea concentration

Was measured with a commercial kit (LABTEST, Brazil) according to the manufacturer’s instructions.

#### Serum and urine creatinine

Creatinine concentrations were determined with the modified Jaffe method^24^ and used to calculate the creatinine clearance according to the following formula: (urine creatinine × urine volume)/(serum creatinine × 1440).

#### Urine oxalate

Was analyzed with an oxalate oxidase kit (Trinity Biotech, Co. Wicklow, Ireland) following the manufacturer’s protocol. The results were corrected through the urine volume and were expressed as mM/24 h.

#### Urine crystals

Urine sediment was obtained by centrifugation (2,300 rpm/min for 5 minutes). Calcium oxalate crystals were photographed (Supplemental material) and counted in a Neubauer chamber following the formula: (final volume × number de crystals)/(initial volume × 0.0001). The results were expressed as urinary crystals/ml.

#### Histochemistry assay

Paraffin sections were subjected to xylene and alcohol gradient solutions, antigen retrieval recovery, protein block, and incubation with primary rabbit anti-TGF-β1 (1:100, Abcam, Cambridge, MA, USA) overnight at 4 °C. After this period, the sections were incubated with a dextran polymer conjugated to peroxidase antibody for 30 minutes (DAKO Envision kit, Dako, Denmark). Marking was detected by exposing the sections using a chromogenic substrate. The primary antibody was omitted as a negative control, and the sections were counterstained with hematoxylin and analyzed under a light microscope the Olympus BX 60 model at 20x or 40x magnification. The paraffin section of the kidney were also stained with sirius red and quantified under a polarized light microscope (Olympus BX 60) to evaluate the production of collagen types (type I yellow to red tone and type III greenish tone). The obtained microscope images were quantified using Image J software and expressed as a percentage of the stained area.

### *In vitro* assay

#### HK2-WT (wild type)

Human proximal tubular epithelial cells (HK2 wild type) were exposed for 48 hours or 72 hours to the control culture medium (DMEM + F12, 5% FBS), oxalate (Ox 0.5 mM of potassium oxalate diluted in phosphate buffered saline (PBS), CaOx crystals (CaOX 50 μg/ml prepared as previously described)^25^ and TGF-β1 (10 ng/ml, Peprotech, NJ, US). In the supplemental material, the cells were exposed to calcium phosphate crystals (CF50 μg/ml and CF100 μg/ml) during 48 h. In some experiments, the cells were subjected to the previously reported groups concomitantly with BMP-7 (HK2 + BMP-7) (50 ng/ml, Peprotech, NJ, US). The experimental protocol was repeated at least 3 times at different periods. N = 15 for each group.

#### HK2T (overexpression of BMP-7 in HK2 cells)

The cells HK2 (wild type) (40% confluency) were treated with a mixture of BMP-7-GFP containing a mammalian expression vector (TrueClone Origene, Rockville, US) and liposomes (Lipofectamine, Life Technologies) for six hours. After this period, the cells were selected through antibiotic resistance (neomycin 400 μg/ml). GFP-positive cells that overexpressed BMP-7 were selected and exposed to the previously reported groups.

#### HK2T + siRNA (BMP-7 silencing by small interfering RNA)

HK2 cells overexpressing BMP-7 (HK2T) were treated with a mixture of siRNA for human BMP-7 (FlexiTube, Qiagen, Hilden, Germany) and liposomes in accordance with the manufacturer’s instructions. Cells that were transfected with scrambled siRNA served as the controls. Six hours after the transfection, the cells were incubated with oxalate (0.5 mM), CaOx crystals (50 μg/ml), and TGF-β1 (10 ng/ml) for 48 h to observe the effects of BMP-7 silencing.

#### Quantitative Real-time PCR

The total RNA was purified from cells with the phenol and guanidine isothiocyanate-cesium chloride method using an appropriate kit (Trizol, Life Technologies, USA) and reverse transcribed into cDNA (High Capacity cDNA Reverse Transcription, Applied Biosystems, CA, USA). Real-time amplification was obtained using a GeneAmp 7700 Sequence Detection System (SDS, ABI Prism 7700, Applied Biosystems) and monitored using the SYBR Green I intercalating dye (Applied Biosystems). PCR was performed with selective primers for E-cadherin (Forward: TGCTGCAGGTCTCCTCTTGG; Reverse: AGTCCCAGGCGTAGACCAAG), Cytokeratin (Forward: ACAATTTGTCTGCCTCCAAGGTCC; Reverse: TCTACCCAGAAGACACCCTCCAAA), smooth muscle α-actin (Forward: TTGCTGACAGGATGCAGAAGGAGA; Reverse: ATCTGCTGGAAGGTAGACAGCG), TGF-β1 (Forward: TGGACACCAACTATTGCTTCAGCTCC; Reverse: GAGGTCCTTGCGGAAGTCAATGTA), β-actin (Forward: TTTGAATGATGAGCCTTCGTGCCC; Reverse: CAAGTCAGTGTACAGGTAAGCCCT) and Vimentin (Forward: ATGGCCCTTGACATTGAGATTGCC; Reverse: TGGGTATCAACCAGAGGGAGTGAA) (Supplemental material). The results of fifteen experiments per group were reported as a relative expression normalized with the β-actin housekeeping gene. The fold variation was determined using the 2^ΔΔCt^ method according to previously published protocol^26^.

#### Immunofluorescence

The cells were grown on Labtek II glass slides, washed with PBS, fixed (3.7% fresh paraformaldehyde in PBS for 15 minutes at room temperature), permeabilized (0.2% Triton X-100 for 5 minutes), and blocked with 0.5% BSA for 1 h. After this period, the cells were incubated with the primary antibodies at a ratio of 1:50 to E-cadherin, smooth muscle α-actin, Cytokeratin, Vimentin (Supplemental material, Sigma-Aldrich, MO, USA) and TGF-β1 (Abcam, Cambridge, MA, USA), overnight at 4 °C. The FITC or TRITC-labeled secondary IgG (1:100) (Santa Cruz Biotechnology, Dallas, TX, USA) was added for 1 h, and then the cells were incubated with DAPI (4′6-diamidino-2-phenylindole, Sigma) 10 μg/ml. Coverslips were mounted on the glass slides using glycerin, and the slides were analyzed using microscopy at 40x magnification. The microscope images obtained were quantified using Image J software and expressed as fluorescence intensity (pixel).

#### Western Blotting

Cells and kidney tissues were lysed with a 200-μL RIPA lysis buffer per plate (100 mm^2^). The lysates were centrifuged at 12,000 g for 5 min at 4 °C, and the supernatants were stored at −80 °C. Proteins (30 μg) were separated by 10% polyacrylamide gel electrophoresis and transferred to polyvinylidene fluoride (PVDF) membranes using a Mini Trans-Blot Electrophoretic Transfer Cell (BioRad). Nonspecific binding sites were blocked with 5% albumin (v/v) in a TBS buffer. The immunoblots were incubated overnight at 4 °C with the TGF-β1 or GAPDH primary antibodies (1:1000, Santa Cruz Biotechnology, Dallas, TX, USA). After washing three times with TBS-T, the membranes were incubated for 1 h at 4 °C in HRP-conjugated secondary antibodies (1:30000; Cell Signalling). Immunoreactive protein bands were visualized using Pierce ECL Plus Chemiluminescent substrate detecting reagents (Thermo Fisher, USA). Images were obtained and analyzed with an Alliance 7 Chemiluminescence documentation system (UVItec, Cambridge, UK). The immunoblot band intensities were quantified using Image J software and expressed as the TGF-β1/GAPDH ratio.

#### Invasion assay

Cell invasion was analyzed using a trans-well assay. Cells (10^5^) were added to the top chambers of six-well trans-well plates (8 μm size, Millipore Corporation, Bilerica, MA, USA) covered with a matrigel matrix (200 μg/ml, Corning, USA). Culture media containing 5% FBS Ox (0.5 mM), CaOx (50 μg/ml), or TGF-β1 (10 ng/ml) were added to the bottom chambers for 48 h. Top (non-invasive) cells were removed, and bottom (invasive) cells were fixed in 3.7% formaldehyde, stained with Toluidine Blue, and solubilized in SDS (2%); the absorbance was recorded at 520 nm. The results were presented as the average absorbance of invasive cells.

#### Migration assay

The cells were grown to confluence in plastic culture dishes. After 24 h of quiescence, the cells were denuded by dragging a rubber policeman through the center of the plate. The cultures were replaced with fresh media containing 5% FBS (control condition); media containing Ox (0.5 mM), CaOx (50 μg/ml), or TGF-β1 (10 ng/ml) was added, and photographs were taken after 48 h.

#### Statistical analyses

The results were expressed as mean ± SEM. The statistical analysis was performed using one-way analysis of variance (ANOVA) followed by a post-hoc Tukey’s test or Student’s t-test. p-values < 0.05 were considered statistically significant.

## Results

Hyperoxaluria and crystalluria can be induced by the administration of agents such as hydroxy-L-proline (HLP) or ethylene glycol (ETG) in food or water, resulting in chronic renal disease. Recent studies using models in rats and mice indicate that the renal deposition of CaOx crystals occurs mainly in the interstitial region and may be linked to the process of injury and inflammation^24^.

**Figure 1:**
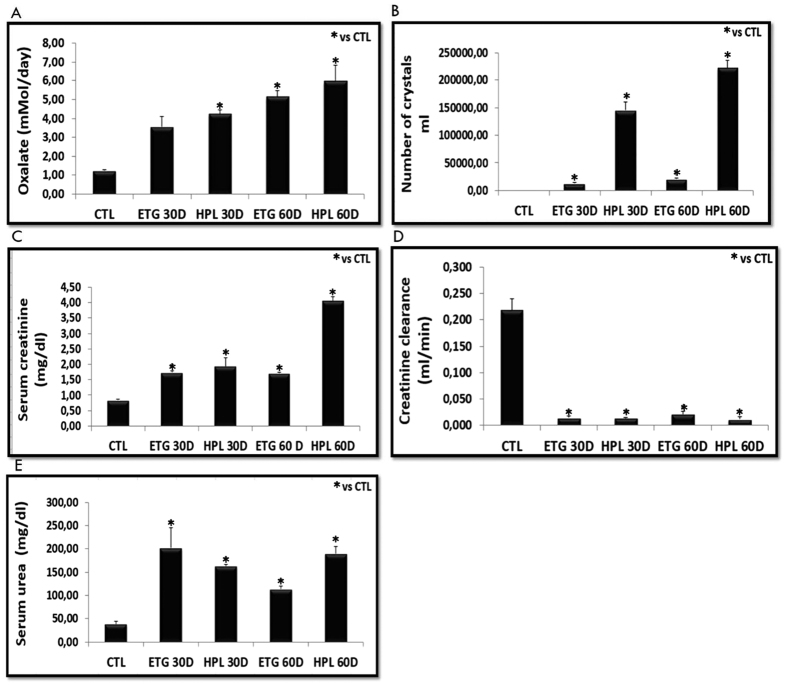
Animals were stimulated with hydroxyproline (HPL) and ethylene glycol (ETG) during 30 (30D) or 60 (60D) days increased oxalate excretion (**A**) and increased urinary crystals number (Neubauer camera count) (**B**). Impaired renal function has been demonstrated through increased serum creatinine (**C**), decreased of creatinine clearance (**D**), and increased serum urea (**E**). Control values represent the average of the data for the control group. Data are presented as means ± standard errors. N = 5 for each group. (*) Indicates significant differences compared with the control group at p < 0.05.

The results of our *in vivo* hyperoxaluric models are shown in Fig. 1. Animals fed with HPL or ETG for 30 or 60 days had an increased excretion of Ox and CaOx crystals in their urine (Fig. 1A and B, Supplemental Fig. 1). We also observed an increase in serum creatinine (Fig. 1C), decrease in creatinine clearance (Fig. 1D), and an increase in serum urea (Fig. 1E) in hyperoxaluric animals compared with the control group. Additionally, the effects were time-dependent (urinary oxalate excretion, crystal excretion) and more prominent in the HPL-treated animals. Our results demonstrate that hyperoxaluria significantly impaired kidney function in mice.

**Figure 2:**
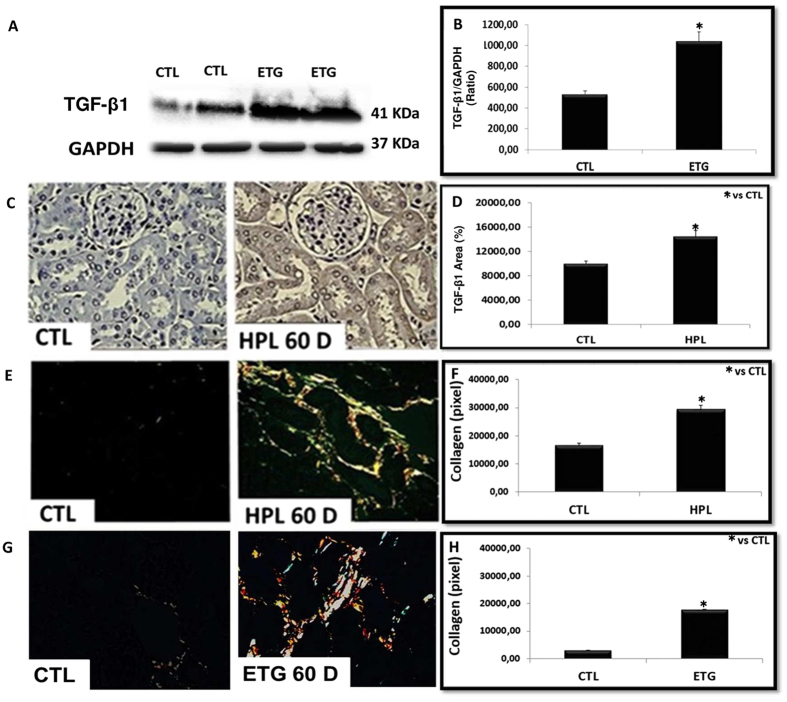
Hyperoxaluria increased the expression of renal fibrosis markers. The stimulation with ethylene glycol during 60 days (ETG 60 D) and hydroxyproline during 60 days (HPL 60 D) increased the expression of TGF-β1 according to immunoblot image (**A**) and percentage of immunostaining area (**C**). There was an increase in the production of collagen type I (yellow to red tone) and collagen type III (greenish tone), showed by picrosirius red staining (**E** and **G**). Densitometric quantification of western blot bands (**B**), immunohistochemistry positive area (**D**) and collagen staining by picrosirius red (**F** and **H**) using ImageJ software. There was a significant increase in TGF-β1 protein expression and collagen production in HPL and ETG treated animals in comparison to controls (CTL) animals. Data are presented as means ± standard errors. N = 5 for each group. (*) Indicates significant differences compared with the control group at p < 0.05.

The expression of TGF-β1 (Fig. 2A and C) and the deposition of collagen fibers (type I yellow to red tone and type III greenish tone) (Fig. 2E and G) are shown in Fig. 2. Hyperoxaluric mice treated with HPL or ETG increased TGF-β1 expression (Fig. 2A–D) and collagen I and III fibers (Fig. 2E–H) in comparison to the control animals.

Our *in vivo* results suggest that Ox and CaOx crystals can induce kidney injury with collagen deposition in our experimental model of hyperoxaluria induced by HPL or ETG. The main mediator of fibrosis is TGF-β1. This mediator can generate myofibroblasts from the tubular epithelial cells through EMT, and BMP-7 can be used to blunt it. The Ox and CaOx capacity to induce EMT was observed *in vitro*.

**Figure 3:**
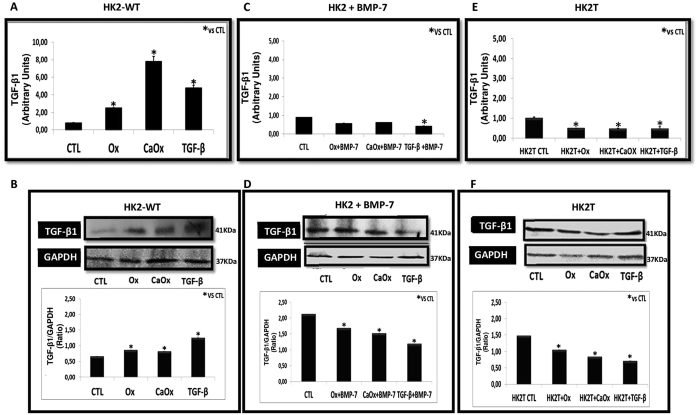
Wild type HK2 (HK2-WT) cells stimulated with potassium oxalate (Ox), calcium oxalate (CaOx), and TGF-β1 increased expression of TGF-β1 (**A** and **B**), while HK2 cells receiving BMP-7 (HK2 + BMP-7) (**C** and **D**) and HK2 overexpressing BMP-7 (HK2T) (**E** and **F**) did not increase TGF-β1 expression when compared to the control situation. Quantitative PCR analysis showing TGF-β1 mRNA expression in comparison to control situation (**A**,**C** and **E**). Western blot analysis and densitometric quantification using ImageJ software showing TGF-β1 protein expression in comparison to controls (CTL) (**B**,**D** and **F**). Data are presented as means ± standard errors. N = 15 for each group. (*) Indicates significant differences compared with the control group at p < 0.05.

Figure 3 shows the expression of TGF-β1 induced by Ox (0.5 mM), CaOx (50 μg/ml) and exogenous TGF-β1 (10 ng/ml). Our results revealed that there was a significant increase in TGF-β1 expression (Fig. 3A) and protein synthesis (Fig. 3B) due to Ox, CaOx, and TGF-β1 in HK2-WT. Additionally, it is interesting to note that in our experimental conditions TGF-β1 was able to stimulate its gene expression and amplify its signal in a “positive feedback” loop.

This increase was blunted in cells previously treated with exogenous BMP-7 (HK2 + BMP-7) (Fig. 3C and D) or transfected with BMP-7 (HK2T cells) (Fig. 3E and F). The TGF-β1 synthesis decreased in HK2 + BMP-7 or HK2T cell groups when stimulated with Ox, CaOx, and TGF-β1.

**Figure 4:**
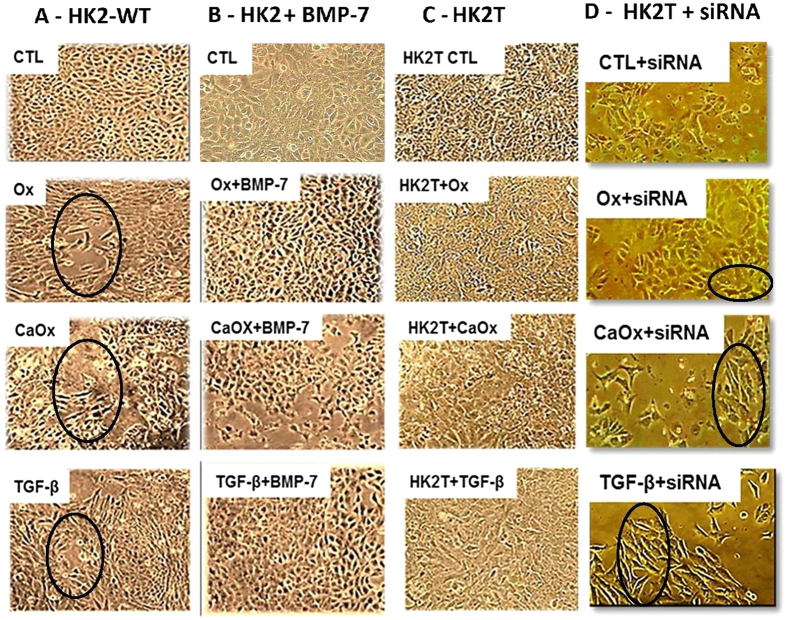
Epithelial cell culture showing monolayer growth with clear and rounded delimitations. On the contrary, mesenchymal cells lose cellular adhesion and present long, tuned and scattered morphology (black circle). Representative light microscopy images showing phenotypical changes in the HK2-WT (**A**), HK2 treated previously with BMP-7 (**B**), HK2 overexpressing BMP-7 (HK2T) (**C**) and HK2T and instable transfected with BMP-7 siRNA (HK2T + siRNA) (**D**). HK2 cells transfected or not were stimulated with potassium oxalate (Ox), calcium oxalate (CaOx) and TGF-β1 and compared to HK2 in control situation (CTL). On the contrary, HK2 + BMP-7 or HK2T showed only few or no morphological differentiations even in the presence of Ox, CaOx, and TGF-β1 in comparison to controls (CTL) situation. N = 15 for each group.

Figure 4 displays the phenotypic characteristics of HK2 cells wild type (HK2-WT, column A), treated with exogenous BMP-7 (HK2 + BMP-7, column B), transfected with BMP-7 gene (HK2T, column C) and HK2T unstable transfected with BMP-7 (HK2T + siRNA, column D) exposed to Ox, CaOx, and TGF-β1. The stimulation with Ox, CaOx and TGF-β1 significantly changed the phenotypic characteristics of HK2-WT in comparison to control situation (Column A circled area). The control cells had a very clear and round boundary with individual cells abutting each other in a uniform array. There were also adhesions between neighboring cells. The stimulation of tubular proximal cells with Ox, CaOx and TGF-β1 induced significant morphological changes in these cells from a cobblestone-like monolayer of epithelial cells to the dispersed, spindle-shaped cells with migratory protrusions. In contrast, in HK2 cells that had been previously treated with BMP-7 (HK2 + BMP-7) or HK2T (Column B and C), only a few morphological differentiations were observed, even in the presence of Ox, CaOx and TGF-β1. This result suggests that the overexpression or the treatment with BMP-7 was effective at inhibit the mesenchymal phenotype induction. Additionally, when HK2T cells were unstable transfected with BMP-7 siRNA, blunting BMP-7 overexpression, they were able to change their morphology in the presence of Ox, CaOx and TGF-β1 (Column D, circled area).

**Figure 5:**
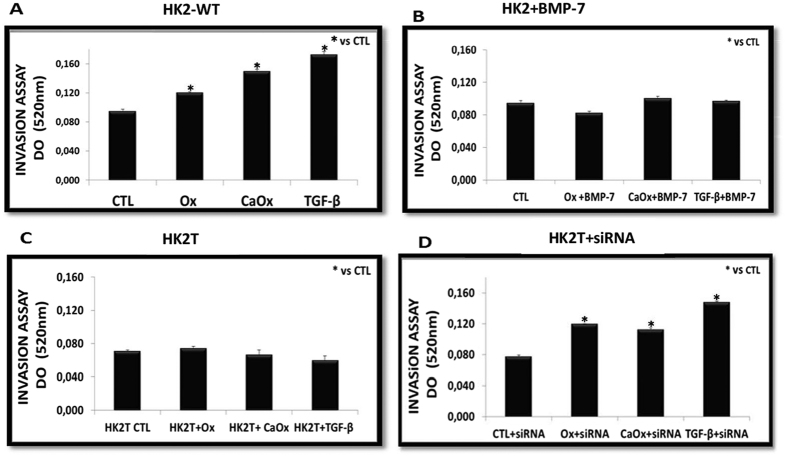
As a result of loss of cell adhesion and morphological changes from cuboid to fusiform form, these cells acquire the ability to invade (transwell chamber assays) and move in the extracellular matrix. The HK2-WT (**A**) and HK2T + siRNA (**D**) acquired invasive capacity, while HK2 cells receiving BMP-7 (HK2 + BMP-7) (**B**), and HK2 cells overexpressing BMP-7 (HK2T) (**C**) behaved similarly the control (CTL) situation even in cell culture exposed to potassium oxalate (Ox), calcium oxalate (CaOx) and TGF-β1. Results were expressed as optical density (OD) and (*) groups were significantly different from the control situation (p < 0.05). N = 15 for each group.

To determine whether HK2-WT cells exposed to Ox, CaOx, or TGF-β1 became more active in the invasion than in the control cells, we evaluated the invasive ability of cells using a transwell chamber assay. As shown in Fig. 5, Ox, CaOx, and TGF-β1 stimulated the HK2-WT (Fig. 5A) to migrate (cells were fixed and stained with Toluidine Blue) faster than the control cells, as shown by spectrophotometric quantification. Nevertheless, this ability was lost in cells treated with BMP-7 (HK2 + BMP-7, Fig. 5B) or overexpressing BMP-7 (HK2T, Fig. 5C). When BMP-7 overexpression was blunted (Fig. 5D, HK2T + siRNA), the invasive ability in response to Ox, CaOx and TGF-β1 was recovered.

**Figure 6:**
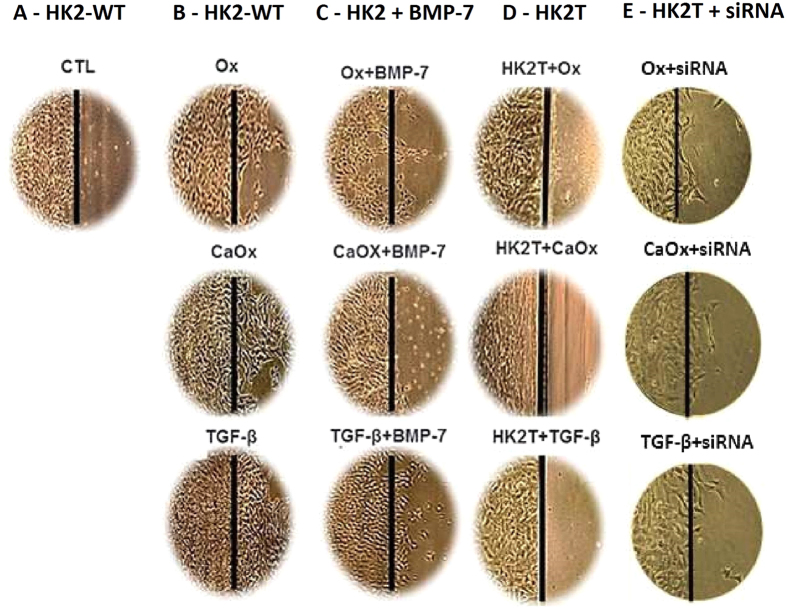
Another characteristic of mesenchymal cell is the acquisition of migration ability. Through light microscopy images we can observe that HK2-WT cells in situation control (**A**), even in confluence, do not migrate to the adjacent region of the culture plate. However stimulated with potassium oxalate (Ox), calcium oxalate (CaOx) and TGF-β1, HK2-WT cells and HK2T + siRNA migrate to the adjacent region (**B** and **E**). However, HK2 cells receiving BMP-7 (HK2 + BMP-7) (**C**) and HK2 cells overexpressing BMP-7 (HK2T) (**D**) behaved similarly to the control (CTL) situation. N = 15 for each group.

The Fig. 6 shows the migration ability through light microscopy images. We can observe that the HK2-WT cells in a control situation (Column A), even confluent, do not migrate to the adjacent denuded region of the culture plate; however, after being stimulated with Ox, CaOx and TGF-β1, we observe the presence of cells in the adjacent region, demonstrating the acquisition of migratory capacity in HK2-WT (Column B). However this ability was decreased in cells treated with BMP-7 (HK2 + BMP-7) (Column C) or overexpressing BMP-7 (HK2T) (Column D). Interestingly, when overexpression of BMP-7 was blunted by siRNA methodology (HK2T + siRNA), the cells recovered migration capability (Column E).

**Figure 7:**
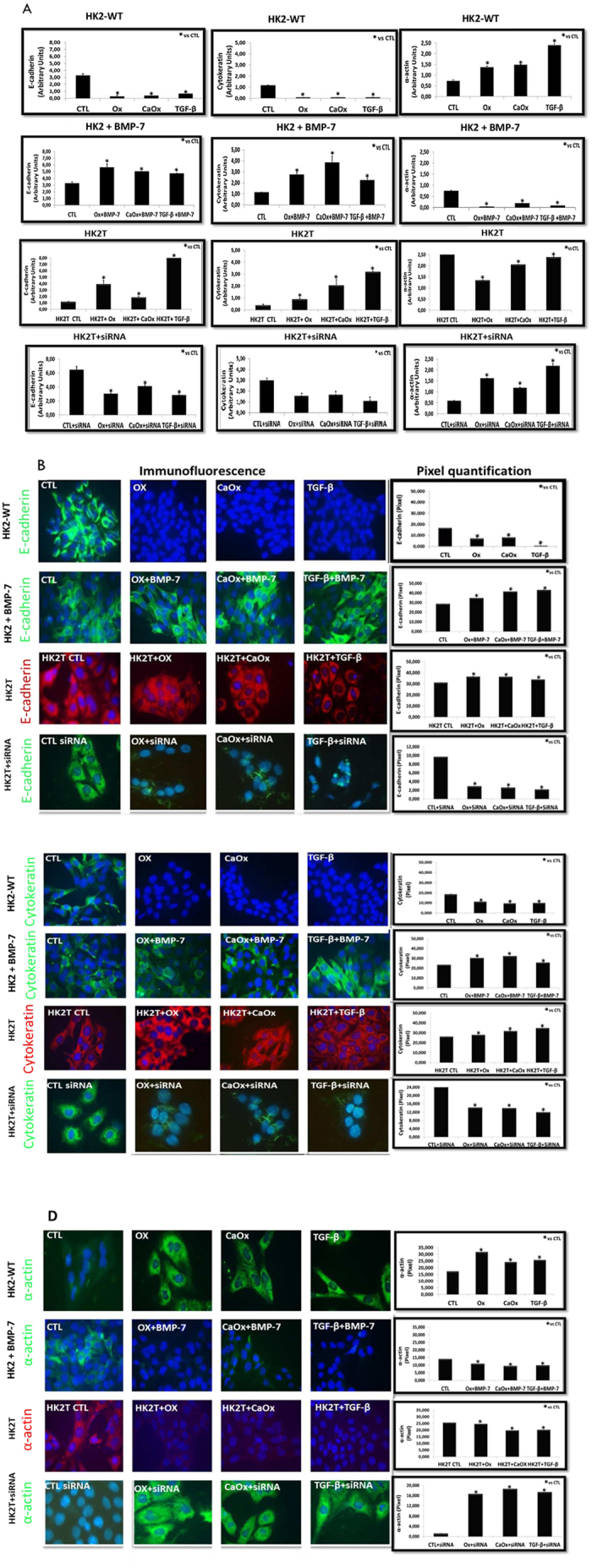
Demonstration of epithelial-to-mesenchymal transition event through epithelial (E-cadherin and Cytokeratin) and mesenchymal markers (smooth muscle α-actin). Quantitative PCR analysis (**A**) and immunofluorescence images (FITC: green fluorescence, TRITC: red fluorescence and blue: nuclei) with their respective densitometric quantification using ImageJ software (**B**,**C**,**D**). We observed an increase in mesenchymal (smooth muscle α-actin) and a decrease in epithelial (cytokeratin and e-cadherin) marker expressions and fluorescence intensity in comparison to the control (CTL) situation in HK2-WT and HK2T + siRNA cells stimulated with potassium oxalate (Ox), calcium oxalate (CaOx) and TGF-β1. On the contrary, in HK2 receiving BMP-7 (HK2 + BMP-7) and HK2 cells overexpressing BMP-7 (HK2T) we did not observe a increase in mesenchymal (smooth muscle α-actin) and a decrease in epithelial (Cytokeratin and E-cadherin) markers. Data are presented as means ± standard errors. N = 15 for each group. (*) Significant different when compared to the control group at p < 0.05.

Figure 7A shows the quantitative analysis of mesenchymal e epithelial cell marker expression. There was a significant increase in mesenchymal marker smooth muscle actin (α-actin) expression and a decrease in epithelial markers expression, including E-cadherin and Cytokeratin, in HK2-WT cells treated with Ox, CaOx, and TGF-β1 compared to the control situation (Fig. 7A). In response to the overexpression of BMP-7 (HK2T) or previous treatment with BMP-7 (HK2 + BMP-7), the epithelial markers did not decrease, on the contrary, increased. The mesenchymal marker smooth muscle α-actin did not increase, on the contrary, decreased when HK2 cells were exposed to Ox, CaOx, or TGF-β1. The immunofluorescence figures and quantifications (Fig. 7B,C and D) corroborated the gene expression results of epithelial and mesenchymal markers. TGF-β1 is the main mediator of epithelial-to-mesenchymal transition. BMP-7 overexpression or treatment, significantly inhibited TGF-β1 expression. This result corroborates the epithelial and mesenchymal markers expression and protein synthesis observed in Fig. 7, showing that BMP-7 blunted EMT.

HK2T exposed to BMP-7 siRNA (HK2T + siRNA) in the presence of CaOx, Ox, and TGF-β1 showed a decrease in epithelial markers (E-cadherin e Cytokeratin) and an increase in mesenchymal marker smooth muscle α-actin (Fig. 7A and B) in comparison to the control experimental situation.

Our results demonstrate that Ox and CaOx can induce EMT *in vitro*, and this effect can be blocked by BMP-7 and this effect is reversible.

Additionally, vimentin is another mesenchymal marker and Ox, CaOx and TGF-β1 exposition during 48 h increased vimentin expression and protein synthesis (Supplemental Fig. 3).

## Discussion

The pathophysiology of tubulointerstitial fibrosis results from cell damage, migration of mononuclear cells into the interstitium, maturating into macrophages, and activating myofibroblasts. The soluble products secreted by these cells contribute to inflammation, the accumulation of an extracellular matrix, and fibrosis. The excessive accumulation of extracellular matrix compromises the functioning of the tubules and peritubular capillaries, resulting in a continuous reduction in glomerular filtration^27^.

Hyperoxaluria or the renal deposition of CaOx crystals mainly in the interstitial region may be linked to the process of injury and inflammation^28^.

In renal tissue hyperoxaluric animals, there was an increase in the production of TGF-β1 and collagen type I and III. The most striking feature of tubulointerstitial fibrosis is the excessive deposition of extracellular matrix, particularly for types I and III collagen fibers^29^,^30^. Under these experimental conditions, we observed increased creatinine and serum urea and a decreased creatinine clearance. This reduction can have several causes; in our case, it was due to tubular damage induced by Ox and CaOx crystal, leading to hemodynamic changes that decreased glomerular filtration.

Studies have reported that EMT plays a crucial role in diverse models of renal fibrosis^31^,^32^. Our study was therefore driven by the hypothesis that Ox and CaOx could induce epithelial mesenchymal transition in renal epithelial cells and thus contribute to the development of renal fibrosis. In this study, we observed that the Ox, CaOx crystals, and TGF-β1 (used as positive control) were efficient in inducing EMT in renal epithelial cells.

This phenotypic transition starts with the loss of apical-basal polarity, which allows mixing between apical and basolateral membrane elements. Additional junctions, including cell-cell adherent junctions and gap junctions, begin to separate, degrading the underlying basement membrane. The expression of E-cadherin is necessary to maintain epithelial integrity; it is responsible for cell-to-cell adhesion. The loss or reduced expression of the adhesion molecule allows the cells to detach more easily from each other. In addition, cytoskeletal components are rearranged.

Cytokeratin is an intermediate filament that acts as a cell scaffold and contributes to the shape and structural integrity of the epithelial cells. Despite resistance, they are dynamic and can rearrange in response to cellular stimuli. During the transformation from epithelial to mesenchymal cells, the replacement of the cytokeratin intermediate filament to the vimentin intermediate filament is observed. A reorganization of the actin cytoskeleton, which favors the acquisition of cell motility, is also likely. Together, these changes transform cells of a cuboidal shape to a fusiform one; finally, the cells acquire the ability to invade and move in the extracellular matrix, since they are devoid of any cell-to-cell contact^33^,^34^.

In this study, we observed that the Ox, CaOx, and TGF-β1 (positive control) were efficient in inducing EMT in renal epithelial cells. This effect was observed after 48 h and 72 h exposition (Supplemental Fig. 2), showing time dependence and was specific for Ox and CaOx, since in our experimental conditions, calcium phosphate did not induce TGF-β1 production (Supplemental Fig. 3). There was also a phenotypic change; the cells acquire mesenchymal characteristics, including down-regulation of epithelial markers: E-cadherin and cytokeratin resulting to disintegration and loss of the cell contact, the cells acquired both migration and invasion ability. On the other hand, the cells undergoing EMT up-regulates mesenchymal markers: smooth muscle α-actin. In our experimental conditions, the mesenchymal marker vimentin was also upregulated (Supplemental Fig. 3).

Several previous studies have described the exposure of macrophages, the main cell producing TGF-β1, to CaOx induced epithelial mesenchymal transition in tubular cells^10^. However, ours is the first study to show that the proximal tubule cells can be subjected to EMT after direct exposure to the main components of kidney stones.

TGF-β1 has been implicated as a key molecule in fibrosis and immune regulation, and its expression is increased in numerous fibrotic conditions^35^. In our analysis, there was an increase in the TGF-β1 expression in cells stimulated by Ox, CaOx, and TGF-β1. Interestingly, the group that received treatment with TGF-β1 induced their own synthesis in a manner similar to a positive feedback loop. Its ability to stimulate production has been demonstrated by other authors in previously published studies^36^.

Several researchers have attempted to reverse renal fibrosis by testing antifibrotic substances. BMP-7 therefore emerges from counteracting the effects of fibrotic TGF-β1^18^,^21^. TGF-β1 promotes the phosphorylation of Smads 2 and 3, while BMP-7 promotes phosphorylation of Smads 1, 5, and 8. Both complexes have been associated with Smad4 (Co-Smad), which translocate to the nucleus to induce the direct transcription of various target genes, demonstrating that BMP-7 or TGF-β1 effects are both excludents^18^.

Luo D *et al*.^13^ demonstrated that BMP-7 can prevent the TGF-β1 expression or increase the expression for the SnoN protein, which is turn is a transcriptional repressor via Smad 3.

We also observed that cells stimulated with Ox, CaOx, and TGF-β1 and concomitantly treated or transfected with BMP-7 showed a blockage evidenced by the decreased expression of TGF-β1 and epithelial markers; conversely, the increased expression of mesenchymal marker. The cells displayed an epithelial cell morphology and did not acquire migration and invasion capabilities, showing the blunt of EMT induced by Ox, CaOx or TGF-β1.

In order to examine if BMP-7 blocks EMT, we arranged the overexpression of BMP-7 and blocked it using siRNA methodology and conducted the EMT characterization experiments. Our results showed that the cells acquired mesenchymal phenotype, increased mesenchymal marker expression, decreased epithelial markers expression and acquisition of cell invasion and migration capacity.

Our results indicated that the treatment of tubular epithelial cells with exogenous BMP-7 or its overexpression were both effective at maintaining the phenotype of these epithelial cells.

Our results support the hypothesis that hyperoxaluric mice showed a decrease in renal function and increase in CaOx crystals and Ox urinary excretion and collagen deposition in the kidney. Our *in vitro* results suggests that EMT can be involved in this effect because Ox or CaOx crystals induced TGF-β1 production and EMT in proximal tubular cells. These cells acquire a myofibroblast phenotype, a collagen producer cell. This effect can be prevented by BMP-7 and silencing of the BMP-7 gene expression in HK2 cells returned the cells to be vulnerable to EMT.

## Additional Information

**How to cite this article**: Convento, M. B. *et al*. Calcium oxalate crystals and oxalate induce an epithelial-to-mesenchymal transition in the proximal tubular epithelial cells: Contribution to oxalate kidney injury. *Sci. Rep.* **7**, 45740; doi: 10.1038/srep45740 (2017).

**Publisher's note:** Springer Nature remains neutral with regard to jurisdictional claims in published maps and institutional affiliations.

## Supplementary Material

Supplementary Dataset 1
