# Supplementary material for: Calcium oxalate crystals and oxalate induce an epithelial-to-mesenchymal transition in the proximal tubular epithelial cells: Contribution to oxalate kidney injury
Source: Sci Rep. 2017 Apr 7;7:45740. doi: 10.1038/srep45740 (PMC5384284; doi:10.1038/srep45740)
Supplement: Supplementary Dataset 1 [file srep45740-s1.doc]

**Calcium oxalate crystals and oxalate induce an epithelial-to-mesenchymal transition in the proximal tubular epithelial cells: Contribution to oxalate kidney injury**

**Marcia Bastos Convento, Edson Andrade Pessoa, Edgar Cruz, Maria Aparecida da Glória, Nestor Schor, Fernanda Teixeira Borges.**

**Supplemental Figures**

**
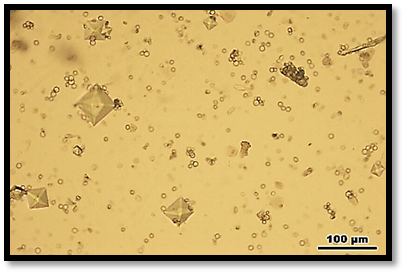
**

**Figure 1**. Light microscopy images showing calcium oxalate crystals observed in the urine of hyperoxaluric animals.


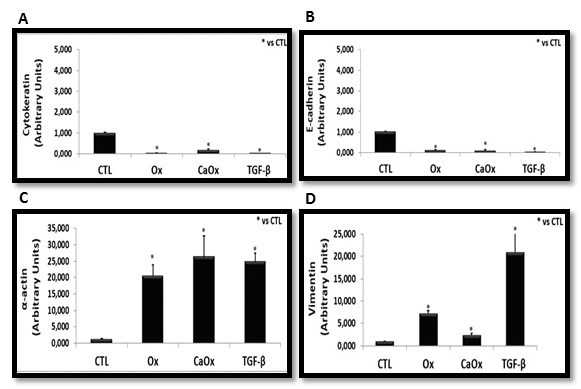


**Figure 2**. Quantitative PCR analysis: demonstration of epithelial-to-mesenchymal transition event in 72 hours through epithelial and mesenchymal markers: Cytokeratin (A), E-cadherin (B), smooth muscle α-actin (C) and Vimentin (D). Data are presented as means ± standard errors. N = 5 for each group. (*) indicates significant differences compared with the control group at p < 0.05


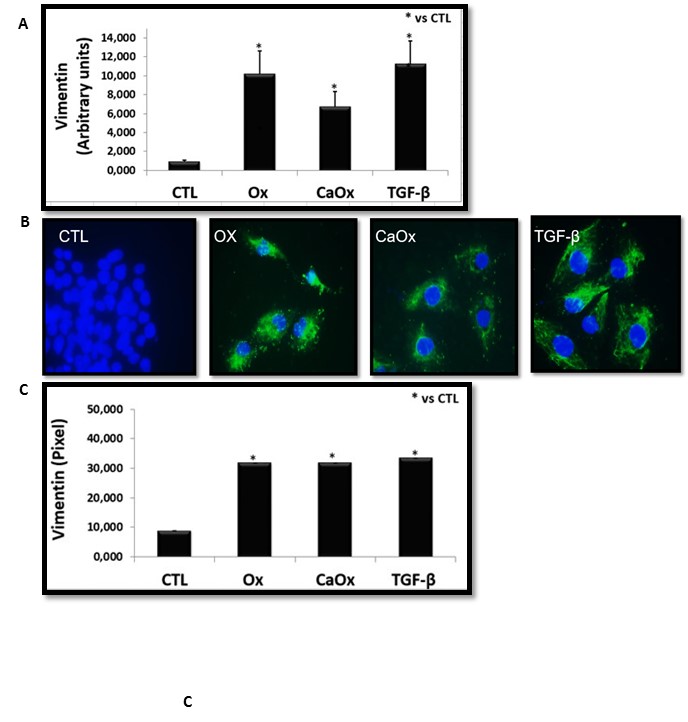


**Figure 3**. Demonstration of epithelial-to-mesenchymal transition event through mesenchymal markers Vimentin in HK2-WT cells (48 hours). Quantitative PCR analysis (A), immunofluorescence images (FITC: green fluorescence and nuclei: blue) (B) with their respective densitometric quantification using ImageJ software (C). Data are presented as means ± standard errors. N = 5 for each group. (*) indicates significant differences compared with the control group at p < 0.05.

**A**

**B** **C**

**Figure 4**. Demonstration of the absence of epithelial-to-mesenchymal transition event in HK2 cells stimulated with calcium phosphate (CF, 50 and 100 µg/ml) during 48 hours. (A) Quantitative PCR analysis through mesenchymal marker smooth muscle α-actin (α-SMA), epithelial marker e-cadherin and EMT inducer TGF-β1. (B) TGF-β1 protein expression by western blot and (C) its respective densitometric quantification using ImageJ software. Data are presented as means ± standard errors. N = 4 for each group.
